# Quercetin Improves Lipopolysaccharide‐Induced Septic Liver Injury by Inhibiting the Activation of ROCK/NF‐κB/NLRP3 Pathway

**DOI:** 10.1002/fsn3.70757

**Published:** 2025-08-04

**Authors:** Lina Xiao, Lingya Kong, Ming Han, Jianing Zhao, Meini Zhang, Yanjin Li, Mingxuan Wang, Zirui Wang, Jiaxuan Li, Zhihong Ma, Zhuojun Deng

**Affiliations:** ^1^ College of Integrative Medicine Hebei University of Chinese Medicine Shijiazhuang Hebei China; ^2^ Emergency Department The Third Hospital of Hebei Medical University (Xiangjiang District) Shijiazhuang Hebei China; ^3^ College of Basic Medicine Hebei University of Chinese Medicine Shijiazhuang Hebei China; ^4^ Hebei International Cooperation Center for Ion Channel Function and Innovative Traditional Chinese Medicine Shijiazhuang Hebei China; ^5^ Hebei Key Laboratory of Integrative Medicine on Liver‐Kidney Patterns Shijiazhuang Hebei China

**Keywords:** lipopolysaccharide, NLRP3 inflammasome, quercetin, rho kinase (ROCK), septic liver injury

## Abstract

Quercetin (QUE) is a common flavonoid compound, found in fruits and vegetables, with powerful anti‐inflammatory and antioxidant properties. Nevertheless, the protective function and mechanisms of QUE in septic liver injury (SLI) caused by lipopolysaccharide (LPS) are still unknown. This study aimed to investigate the effect of QUE on SLI rats and the underlying mechanisms. Forty male Sprague–Dawley (SD) rats were randomly assigned into four groups (*n* = 10 per group): control group (CON), control + QUE group (CON + QUE), LPS group (LPS), and LPS + QUE group (LPS + QUE). Rats were administered intragastrically with QUE or normal saline for one week, and LPS was administered on the last day to induce the sepsis model. The results showed that QUE reduced aspartate aminotransferase (AST) and alanine aminotransferase (ALT) activities in SLI rats (both *p* < 0.05). Additionally, histopathological analysis demonstrated that QUE alleviated the liver lesions in SLI rats. Furthermore, QUE also improved the oxidative stress and inflammatory responses, showing the decrease of malonaldehyde (MDA), tumor necrosis factor (TNF)‐α, interleukin (IL)‐1β, IL‐18, and IL‐6 and the increase of superoxide dismutase (SOD) in SLI rats (all *p* < 0.05). In addition, QUE decreased the expression of Toll‐like receptor 4 (TLR4), phosphorylated myosin phosphatase target subunit (p‐MYPT)‐1, phosphorylated nuclear factor kappa‐B (p‐NF‐κB), NOD‐like receptor family pyrin domain containing 3 (NLRP3), apoptosis‐associated speck‐like protein (ASC), caspase‐1, and cleaved‐caspase‐1 in liver (all *p* < 0.05). These results suggested that QUE could be able to treat SLI partly via stimulating the ROCK/NLRP3 pathway.

## Graphical abstract

- Quercetin has potential protective effects on sepsis liver injury (SLI) induced by lipopolysaccharide.
- Quercetin inhibits activation of the NLRP3 inflammasome in SLI liver.
- Quercetin suppresses Rho kinase/NF‐κB signaling in SLI liver.

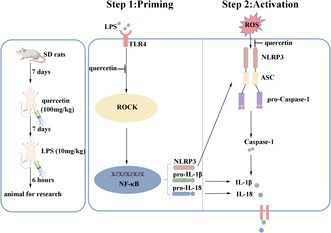

## 1 Introduction

Sepsis is a dysregulated host response to infection, which can lead to fatal multi‐organ dysfunction (Singer et al. 2016). Sepsis can be driven by a variety of pathogens, such as fungi, bacteria, viruses, and parasites. The most frequent cause of sepsis among these is bacteria, particularly gram‐negative bacteria. Lipopolysaccharides (LPS) from the outer membrane of gram‐negative bacteria are the key trigger of sepsis and frequently result in multiple organ damage (Perez‐Hernandez et al. 2021). The liver is one of the earliest occurring and the most susceptible target organs during sepsis. Septic liver injury (SLI) has high mortality and poor prognosis, imposing a heavy medical burden on society. So far, there hasn't been much successful targeted therapy for SLI worldwide. Therefore, it is imperative to explore the precise pathogenesis of SLI and to look for innovative targets and therapeutic medications.

Innate immunity has a significant influence in the pathogenesis of SLI. NOD‐like receptor family pyrin domain containing 3 (NLRP3) inflammasome is a multiprotein complex that is crucial to both innate immunity and the development of many inflammatory diseases. It is reported that the process of SLI is significantly affected by the NLRP3 inflammasome activation (Li et al. 2022). Priming and activation are the two sequential and necessary stages for the activation of the NLRP3 inflammasome. LPS binds to Toll‐like receptor 4 (TLR4), which causes nuclear factor κB (NF‐κB) to undergo nuclear translocation and sends the initial signal that stimulates the expression of NLRP3, pro‐interleukin (IL)‐1β and pro‐IL‐18. Reactive oxygen species (ROS) produced during sepsis can act as a second signal to stimulate the activation of the NLRP3 inflammasome (Akbal et al. 2022; Xu and Nunez 2023). In summary, the promising therapeutic target for SLI is the suppression of the initiation and activation pathway of the NLRP3 inflammasome.

Rho kinase (ROCK), a well‐known downstream effector of the small guanosine triphosphatases (GTPases), RhoA, B, and C, regulates a wide variety of biological processes that enable tissues to respond to stress and injury as vital coordinators (Zhang et al. 2020). Some studies have revealed that the RhoA/ROCK signaling pathway is activated by infection, inflammation, and stress, and induces NF‐κB activation, which intensifies the body's inflammatory response (Lu et al. 2024). ROCK inhibitors have been discovered to ameliorate LPS‐induced liver injury (Ding et al. 2016; UludaĞ et al. 2021) and acute lung injury (Liu, Pan, et al. 2022). Therefore, reducing the expression of RhoA/ROCK seems to be a useful therapeutic method for alleviating organ damage in sepsis and offers a fresh idea on how to handle SLI.

**FIGURE 1:**
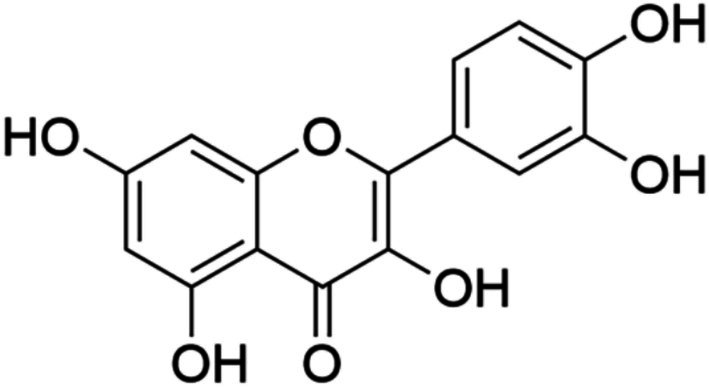
The general structure of quercetin.

Many substances sourced from plants such as baicalein and rosmarinic acid have been proven to have a protective effect on organ damage induced by sepsis (Dicle et al. 2024; Jiang et al. 2009). Quercetin (QUE) (Figure 1) is the major representative of the flavonoid compound found in fruits and vegetables, which has strong anti‐inflammatory and antioxidant properties (Vollmannova et al. 2024). QUE exhibits multi‐organ protective properties by modulating various signaling pathways including MAPK/ERK and NF‐κB (Seker et al. 2024). QUE has beneficial effects in various liver diseases such as metabolic associated fatty liver disease, liver cancer (Zhao et al. 2021) and sepsis‐related multiple organ damage (Sang et al. 2022; Peng et al. 2017). In this study, we established a SLI rat model by LPS intraperitoneally and investigated whether QUE improved SLI through the ROCK/NF‐κB/NLRP3 signaling pathway. Our data suggest that QUE protects against LPS‐induced SLI through inhibiting the ROCK/NF‐κB/NLRP3 signaling pathway. Our findings suggest that QUE could be a therapy for SLI.

## 2 Materials and Methods

### 2.1 Materials

QUE (HY‐18085, purity > 98%) was bought from MedChemExpress Biotechnology Co. Ltd. (New Jersey, USA). LPS (L2880) was received from Sigma‐Aldrich Co. Ltd. (St. Louis, USA). The rabbit anti‐mouse primary antibodies used were as follows: NF‐κB (AF5006, Affinity Biosciences, Jiangsu, China), phosphorylated NF‐κB (p‐NF‐κB) (AF2006, Affinity Biosciences, Jiangsu, China), cleaved‐caspase‐1 (AF4022, Affinity Biosciences, Jiangsu, China), caspase‐1 (AF5418, Affinity Biosciences, Jiangsu, China), TLR4 (35,463, Signalway Antibody, Wuhan, China), NLRP3 (30109‐1‐AP, Proteintech Group, Wuhan, China), myosin phosphatase target subunit (MYPT)‐1 (BS1557, Bioworld, Nanjing, China), phosphorylated MYPT (p‐MYPT)‐1 (BS64148, Bioworld, Nanjing, China), Apoptosis‐associated speck‐like protein (ASC) (YT0365, ImmunoWay, California, USA), Glyceraldehyde‐3‐phosphate dehydrogenase (GAPDH) (52902, Signalway Antibody, Wuhan, China), β‐actin (AP0060, Bioworld, Nanjing, China). The secondary antibodies used were as follows: HRP‐labeled goat anti‐rabbit IgG (H + L) (S0001, Affinity Biosciences, Jiangsu, China).

### 2.2 Animals and Ethical Approval

The male specified pathogen‐free (SPF) Sprague–Dawley (SD) rats (6–8 weeks old, weight = 220–240 g) were provided by SPF (Beijing) Bioscience Co. Ltd. (Beijing, China) [Certificate No. SCXK (Jing) 2019–0010]. The rats were bred in the Experimental Animal Center of Hebei University of Chinese Medicine. They were kept at 24°C ± 2°C, housed in standard cages, and had unlimited access to water and food. The research's animal investigations were approved by the Hebei University of Chinese Medicine's Animal Care and Ethical Committee (approval number: DWLL202402041). All methods in this study were completed according to the Regulations on the Administration of Experimental Animals Issued by the State Council of the People's Republic of China (revised 2017).

### 2.3 Experimental Design

After seven days of acclimatization, 40 male SD rats (*n* = 10) were randomly assigned to four groups: control group (CON), control + QUE group (CON + QUE), LPS group (LPS), and LPS + QUE group (LPS + QUE). Rats in the LPS + QUE and CON + QUE groups were administered by gavage at doses of QUE (50 mg/kg/day), while the others were administered by gavage with the same volume of normal saline. The dose of QUE was based on references (Meng et al. 2016). Rats in the LPS group and LPS + QUE groups received 10 mg/kg LPS intraperitoneally on the seventh day, whereas the other rats received normal saline intraperitoneally.

After 6 h of treating with LPS, rats were injected with pentobarbital (40 mg/kg) intraperitoneally. After femoral artery blood was taken, euthanasia was performed by cervical dislocation. The serum was left standing at room temperature for 2 h, and then the supernatant was taken after centrifugation. The liver's identical section is stored in 4% paraformaldehyde, and the rest was stored in a refrigerator at −80°C for further testing.

### 2.4 Preparation of Liver Homogenate

Liver tissue from each rat was precisely weighed and crushed in normal saline. The supernatant was taken out for analysis after centrifugation at 2500 rpm for 10 min.

### 2.5 Detection of Biochemical Indicators in Serum and Liver

The activity of serum aspartate aminotransferase (AST) (C010‐2‐1), serum alanine aminotransferase (ALT) (C009‐2‐1), serum and liver superoxide dismutase (SOD) (A001‐3) and the content of serum and liver malondialdehyde (MDA) (A003‐1) were measured according to the instructions of kits (Nanjing Jiancheng Bioengineering Institute Co. Ltd., Nanjing, China).

### 2.6 Detection of Inflammatory Cytokines in Serum and Liver

Interleukin (IL)‐1β (ERC007), tumor necrosis factor (TNF)‐α (ERC102a), IL‐18 (ERC010), and IL‐6 (ERC003) in serum and liver homogenates were evaluated by enzyme‐linked immunosorbent assay kits (NeoBioscience Technology Co. Ltd., Shenzhen, China).

### 2.7 Evaluation of Histological Changes in Liver

The liver tissue from each rat was preserved in 4% paraformaldehyde for more than 48 h. After gradient alcohol dehydration and xylene dewaxing, the liver tissues were then embedded in molten paraffin wax. Sections were cut at 4 μm thickness, stained with hematoxylin and eosin (H&E) and observed using a Leica DM4000B microscope (Solms, Germany). Each segment was examined at magnifications of ×200 and ×400.

Dihydroethidium (DHE) probe was used to visualize ROS levels in the liver. The samples were incubated with 10 mM 2′,7′‐dichlorofluorescin diacetate (DCHFDA) and superoxide‐sensitive fluorescent dyes in the dark for 30 min, and then washed three times in washing buffer. Next, the slides were incubated with a DAPI solution in the dark for 10 min. Samples from each group were transferred onto identical slides and analyzed under a fluorescent microscopy (Nikon Eclipse C1, Nikon, Japan). Semi‐quantitative analysis was performed based on the optical density data.

To observe the apoptosis of liver tissue, TUNEL staining was conducted by the manufacturer's instructions with some modification (Servicebio, Wuhan, China). The dewaxed sections of liver tissue were washed with distilled water and treated with protease K for 10 min at 37°C. After washing with phosphate buffered saline (PBS), the sections were treated with TdT solution, incubated with 3% hydrogen peroxide for 5 min at room temperature, and immersed in blocking solution for 60 min at room temperature. After adding DAPI dye solution, the section was left out of the light for 10 min at room temperature. Fluorescent microscopy (Nikon Eclipse C1, Nikon, Japan) was then used to collect images; the cells that showed positive apoptosis were red, and the nucleus was marked blue by DAPI. Semi‐quantitative analysis was performed by Image J software 2.14.0 (NIH), and then GraphPad Prism 9.0.0 (San Diego, USA) was selected for data analysis and graphing based on the optical density data.

Immunohistochemistry (IHC) was performed to detect the expression of p‐NF‐κB in liver tissues. Paraffin‐embedded sections were deparaffinized, subjected to antigen retrieval, and blocked for non‐specific binding. The sections were then incubated with primary antibody at 4°C overnight. On the following day, sections were incubated with HRP‐conjugated secondary antibody, followed by DAB chromogenic detection and hematoxylin counterstaining. The distribution and intensity of the brown‐yellow positive signals were observed under a light microscope.

### 2.8 Western Blot Analysis of Protein Expression in Liver

After removed from the −80°C refrigerator, the liver tissue was cut up with scissors and treated with RIPA lysate (Servicebio Technology Co. Ltd., Wuhan, China), phosphatase and protease inhibitors (Boster Biological Technology Co. Ltd., Wuhan, China). Next, the supernatant was extracted after tissue grinding and centrifugation. After the protein was separated by gel electrophoresis, it was quickly transferred to nitrocellulose (NC) membranes. The membranes were enclosed in 5% skim milk at room temperature for 3 h, and then incubated with the following series of antibodies for an entire night at 4°C: TLR4, p‐NF‐κB, NF‐κB, cleaved‐caspase‐1, caspase‐1, ASC at 1:1000 dilution, NLRP3 at 1:2500 dilution, p‐MYPT‐1, MYPT‐1 at 1:750 dilution, GAPDH at 1:5000 dilution, and β‐actin at 1:10000 dilution, respectively. Following washing of the primary antibody, the second antibody was added and incubated at room temperature for 1 h. Then the membranes were treated with the Super ECL detection reagent (Yeasen Biotechnology Co. Ltd. Shanghai, China). Vision Capt software (Kunming Beijie Technology Co. Ltd. Kunming, China) was used to analyze the obtained bands.

### 2.9 Statistical Analysis

The values are expressed as the mean ± SEM. Data analysis was conducted with the GraphPad Prism 9.0.0 (San Diego, USA). One‐way analysis of variance (ANOVA) and Tukey's post hoc test were used to estimate the differences between groups. *p* < 0.05 was considered statistically significant.

## 3 Results

### 3.1 QUE Was a Promising Hepatoprotective Supplementation for SLI in Rats

**FIGURE 2:**
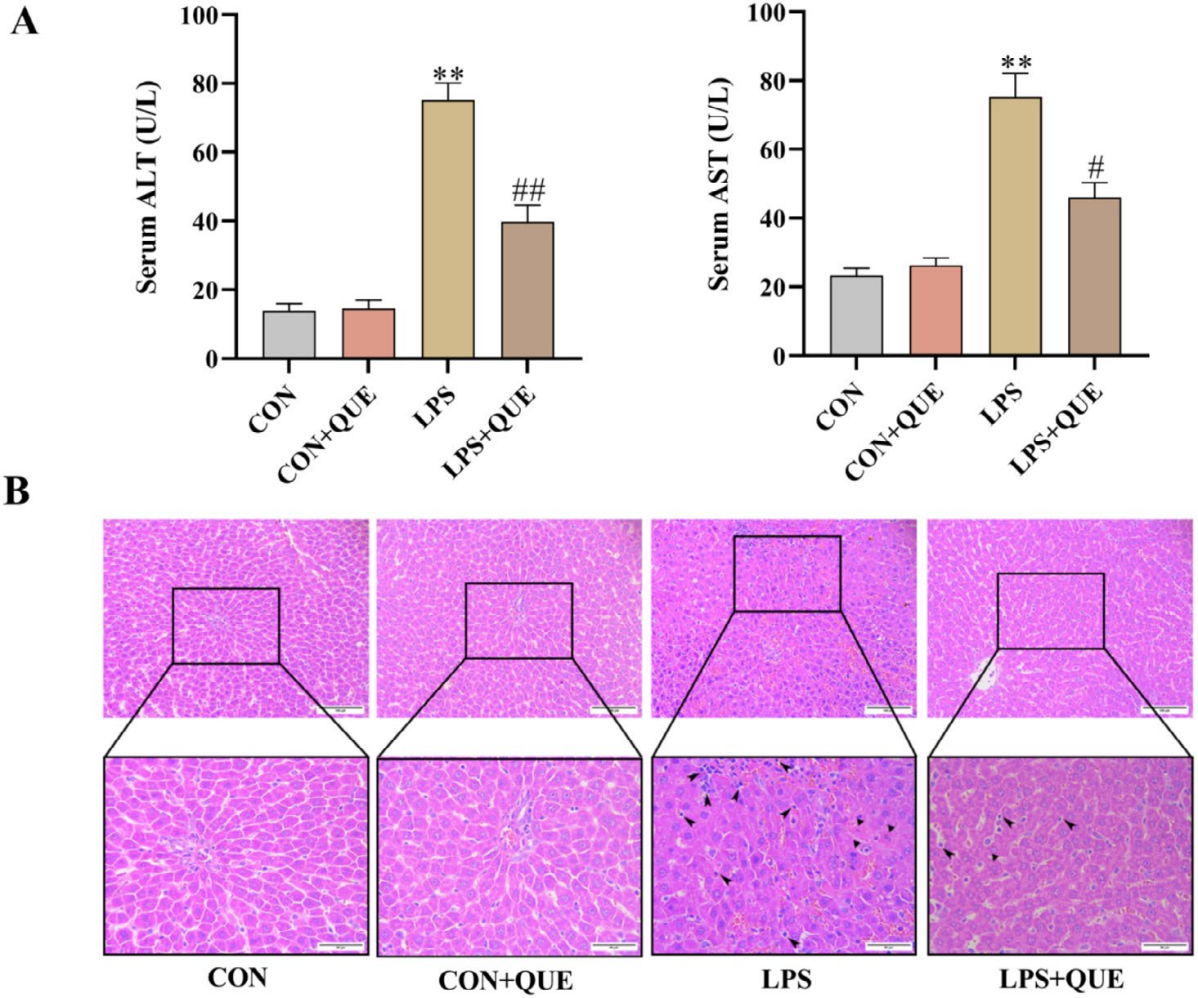
Quercetin had hepatic protection in SLI rats. (A) Serum ALT and AST activity. (B) Representative sections stained with HE (scale bar = 100 μm, 200×, scale bar = 50 μm, 400×). Arrows indicate inflammatory cells, and triangles indicate apoptotic cells. Serum and liver were obtained from the control group (CON), control + QUE group (CON + QUE), LPS group (LPS), and LPS + QUE group (LPS + QUE). Data were expressed as mean ± SEM (*n* = 10). **p < 0.01 and *p < 0.05 versus CON, ##*p* < 0.01 and #*p* < 0.05 versus LPS group.

Serum ALT and AST are two basic indicators of liver damage. ALT and AST activity in the LPS group clearly increased compared to the CON group (both *p* < 0.01), and they decreased in the LPS + QUE group (both *p* < 0.05) (Figure 2A). However, there was no statistical difference between the CON group and the CON + QUE group.

Compared to the CON group, administration of LPS resulted in notable histological alterations in the liver, including hepatocyte degeneration, necrosis, and apoptosis, dilation and congestion of hepatic sinusoids, and infiltration of inflammatory cells; however, QUE pretreatment improved the above histopathological changes (Figure 2B).

### 3.2 QUE Ameliorated the Inflammatory Responses and Oxidative Stress in SLI Rats

**FIGURE 3:**
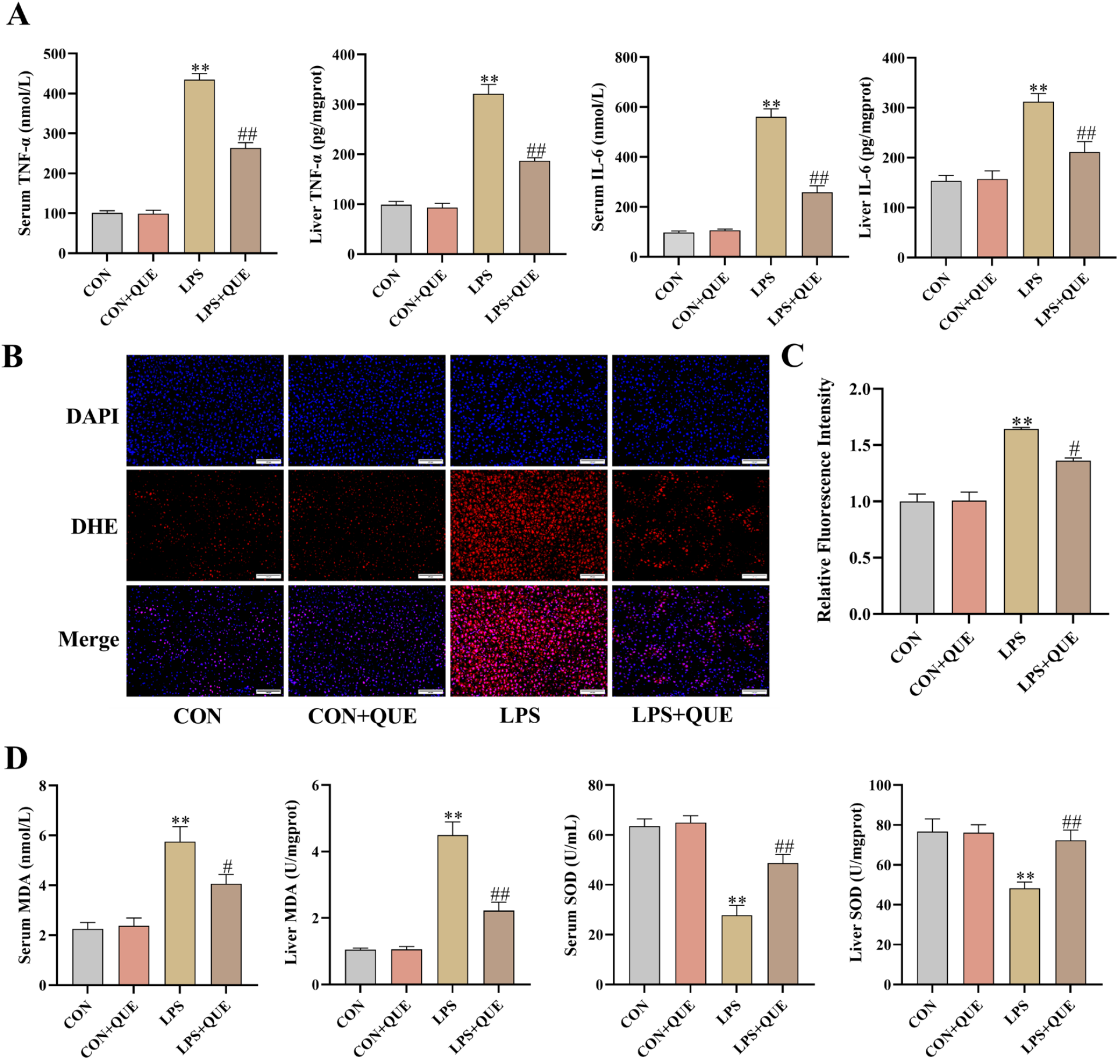
Quercetin ameliorated the inflammatory responses and oxidative stress in SLI rats. (A) Levels of TNF‐α and IL‐6 in the serum and liver. (B) Representative histologic sections of reactive oxygen species (ROS) in four groups (scale bar = 100 μm, 200×). (C) Semi‐quantitative results of ROS. (D) MDA content and SOD activity in the liver and serum. Serum and liver were obtained from the control group (CON), control + QUE group (CON + QUE), LPS group (LPS), and LPS + QUE group (LPS + QUE). Data were expressed as mean ± SEM (*n* = 3–10). **p < 0.01 and *p < 0.05 versus CON, ##*p* < 0.01 and #*p* < 0.05 versus LPS group.

The rats in the LPS group had higher levels of TNF‐α and IL‐6 in serum and liver than in the CON group; however, QUE pretreatment before LPS administration dramatically lowered their levels in comparison to the LPS group (both *p* < 0.01) (Figure 3A).

LPS administration dramatically increased the formation of MDA and ROS levels, and decreased SOD activity in serum and liver tissue compared with the CON group, whereas QUE pretreatment before LPS administration could reverse these changes in comparison to the LPS group (all *p* < 0.05) (Figure 3B,C).

### 3.3 QUE Inhibited the Expression and Activation of Hepatic NLRP3 Inflammasome in SLI Rats

**FIGURE 4:**
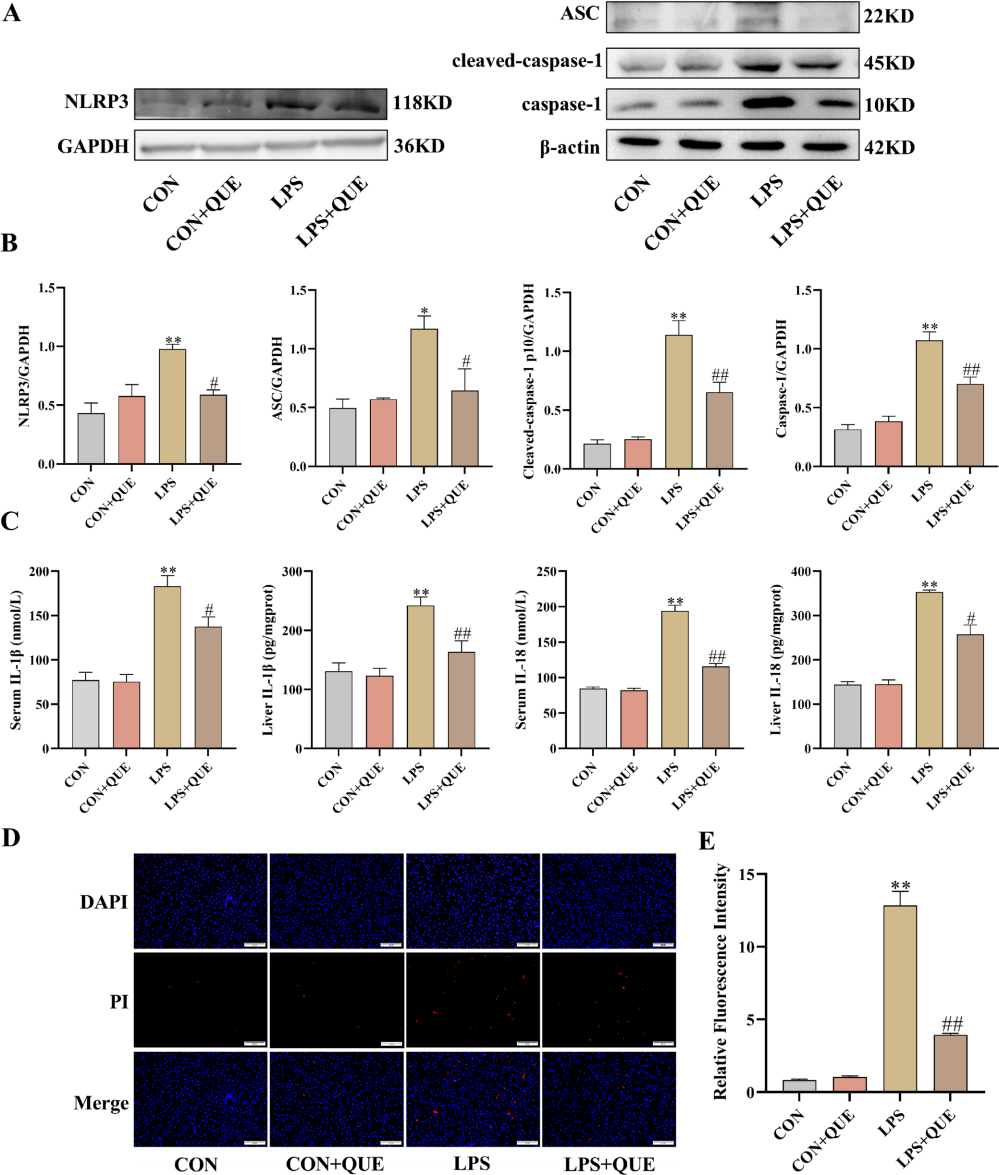
QUE inhibited the expression and activation of NLRP3 inflammasome in SLI rats. (A) Representative expression brands of NLRP3, ASC, caspase‐1, and cleaved caspase‐1. (B) Western blot bands' gray value. (C) Levels of IL‐1β and IL‐18 in the serum and liver. (D) Rat liver tissue stained with TUNEL (200×, scale bar = 100 μm). (E) Semi‐quantitative outcomes of apoptosis rate. Serum and liver were obtained from the control group (CON), control + QUE group (CON + QUE), LPS group (LPS), and LPS + QUE group (LPS + QUE). Data were expressed as mean ± SEM (*n* = 3–10). **p < 0.01 and *p < 0.05 versus CON, ##*p* < 0.01 and #*p* < 0.05 versus LPS group.

When compared to the CON group, the hepatic expression of NLRP3, ASC, caspase‐1, and cleaved caspase‐1 in the LPS group was increased, but QUE pretreatment dramatically reduced the NLRP3, ASC, caspase‐1, and cleaved‐caspase‐1 expression caused by LPS (all *p* < 0.05) (Figure 4A,B).

IL‐1β and IL‐18 are two markers of pyroptosis. They were significantly greater in the LPS group than in the CON group but reduced in the LPS + QUE group (all *p* < 0.05) (Figure 4C). Similarly, the TUNEL‐gray value, which reflects the degree of apoptosis, was significantly elevated in the LPS group compared to the CON group. In contrast, the TUNEL‐gray value was significantly reduced in the LPS + QUE group compared to the LPS group (*p* < 0.01) (Figure 4D).

### 3.4 QUE Suppressed the Expression of TLR4/p‐MYPT‐1/p‐NF‐κB in SLI Rats

**FIGURE 5:**
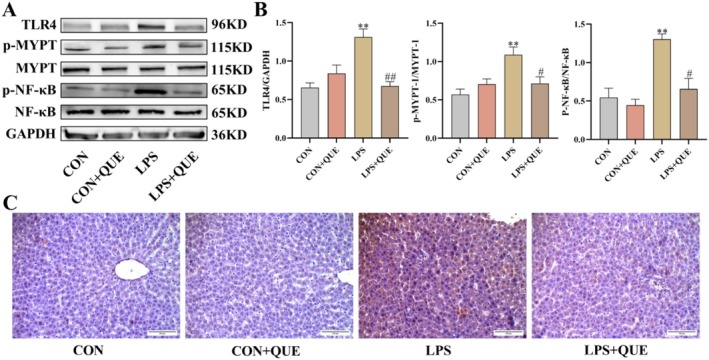
QUE suppressed the expression of TLR4/p‐MYPT/p‐NF‐κB in SLI rats. (A) Typical expression bands for TLR4, p‐MYPT‐1, and p‐NF‐κB. (B) Western blot bands' gray value. (C) Representative IHC images illustrating the nuclear translocation of p‐NF‐κB in liver tissue. Serum and liver were obtained from the control group (CON), control + QUE group (CON + QUE), LPS group (LPS) and LPS + QUE group (LPS + QUE). Data were expressed as mean ± SEM (*n* = 3). **p < 0.01 and *p < 0.05 versus CON, ##*p* < 0.01 and #*p* < 0.05 versus LPS group.

The hepatic protein expression of TLR4, p‐MYPT‐1, and p‐NF‐κB was significantly up‐regulated following LPS administration compared with the CON group; conversely, they were markedly down‐regulated by QUE pretreatment in the LPS + QUE group (all *p* < 0.05) (Figure 5A,B).

The IHC results showed that the expression of p‐NF‐κB markedly increased in the LPS group, evidenced by prominent brown‐yellow granules. It was significantly reduced after QUE pretreatment (Figure 5C).

## 4 Discussion

LPS injection is a simple and direct process that easily causes endotoxemia and inflammation (Kannan et al. 2024); therefore, the LPS‐induced sepsis rat model is currently recognized as an animal model for studying the mechanisms of sepsis (Thomas et al. 2014). In the present study, we demonstrated that QUE significantly alleviates LPS‐induced SLI, potentially through inhibition of the ROCK/NF‐κB/NLRP3 signaling pathway. The SLI model was established by intraperitoneal injection of LPS, showing undesirable alterations in liver function and morphology. Serum ALT and AST in the SLI rats were increased, and hepatic histological analysis with HE staining showed obvious infiltration of inflammatory cells and cell apoptosis. These changes were consistent with the previous study (Aboyoussef et al. 2021). This indicated that we had successfully established a rat model of SLI. QUE pretreatment decreased ALT and AST activities in serum and improved histopathology in liver. This result is consistent with Zhang's report, which confirmed that QUE may be effective in treating SLI (Zhang et al. 2020).

The cytokine storm triggered by the dynamic imbalance of macrophage polarization and the accompanying oxidative stress is the key factor for the progression of sepsis (Ali et al. 2021; Tang et al. 2021; Chen et al. 2021). Cytokine storm is characterized by a significant elevation of inflammatory cytokines including TNF‐α, IL‐6, IL‐18, and IL‐1β. Oxidative stress is an imbalance between the oxidative production and antioxidant capacity, with the excessive accumulation of ROS and MDA, and/or lower activities of antioxidant enzymes such as SOD. Notably, oxidative stress can stimulate the production and release of inflammatory mediators, while the inflammatory response further amplifies oxidative stress, creating a vicious cycle that promotes disease progression. Therefore, one of the strategies for treating SLI is to combat the body's inflammatory responses and oxidative stress. QUE plays a key role in modulating inflammation and promoting tissue repair. QUE inhibits the polarization of pro‐inflammatory M1 macrophages and reduces the release of inflammatory cytokines, thereby alleviating tissue inflammation; on the other hand, it promotes the polarization of anti‐inflammatory and tissue‐repairing M2 macrophages, contributing to the recovery of damaged organs (Seker et al. 2025). In this study, IL‐6, IL‐1β, TNF‐α, IL‐18, and MDA in serum and liver, and ROS in liver tissue raised markedly in SLI rats, accompanied by decreased SOD activity in serum and liver. However, the administration of QUE before LPS injection markedly diminished the levels of inflammatory cytokines, ROS, and MDA, while increasing the SOD activity. Our results were alike with others' reports, which suggested that QUE pretreatment diminished SLI by inhibiting inflammatory responses and oxidative stress (Liu, Liu, et al. 2022). Notably, existing studies have demonstrated that the hepatoprotective effects of QUE are mediated through multiple mechanisms. Specifically, QUE not only binds to the vitamin D receptor (VDR) in liver tissue to regulate hepatic lipid metabolism and alleviate liver inflammation and fibrosis but also targets the RNA‐induced silencing complex (RISC), effectively mitigating liver injury (Sannappa Gowda et al. 2023, 2024; Puttahanumantharayappa et al. 2023). In the future, we will also start from the perspective of VDR to explore its role in SLI.

Innate immune response triggered by LPS is thought to be the essential source of inflammatory responses and oxidative stress in the pathogenesis of sepsis (Miliaraki et al. 2022; Wang et al. 2024). NLRP3 inflammasome plays a crucial role in the innate immune response and inflammatory signals (Blevins et al. 2022). The NLRP3 inflammasome is made up of a sensor (NLRP3), an effector (caspase‐1), and an adapter protein (ASC). NLRP3 is a tripartite protein comprising a C‐terminal leucine‐rich repeat sequence (LRR) domain, a central nucleotide binding and oligomerization domain containing ATPase activity (NACHT domain), and an amino terminal pyrin domain (PYD) (Kelley et al. 2019). The ASC protein has a PYD and a caspase recruitment domain (CARD). When LPS activates the NLRP3 inflammasome, the adapter protein ASC binds to originally inactivated NLRP3 and recruits pro‐caspase‐1. The aggregation of pro‐caspase‐1 leads to its own proteolysis and the production of enzymatically active caspase‐1. Activated caspase‐1 cleaves pro‐IL‐1β and pro‐IL‐18 to mature IL‐1β and IL‐18. As key markers of pyroptosis, IL‐1β and IL‐18 are released during NLRP3 activation. Pyroptosis is characterized by the rupture of the cell membrane and the release of cellular contents, typically accompanied by a strong inflammatory response. The main difference between pyroptosis and apoptosis is that apoptosis occurs through a controlled mechanism without significant inflammation, whereas pyroptosis leads to a strong inflammatory reaction. However, under certain conditions—such as viral infections, bacterial invasion, or severe cellular stress—these two forms of cell death may occur simultaneously or even transition into one another (Yuan et al. 2025). Numerous studies have proven that the activation of the NLRP3 inflammasome is associated with sepsis‐associated organ damage (Swanson et al. 2019). In this research, our findings showed significant increases in the protein expression of NLRP3, ASC, caspase‐1, cleaved caspase‐1, and TUNEL‐gray value in SLI rats. In contrast, the levels of these proteins and TUNEL‐gray value were markedly decreased after QUE pretreatment in SLI rats. These results indicated that QUE could inhibit NLRP3 inflammasome assembly and cell pyroptosis in SLI rats. These findings align with the research carried out by Zhang et al. (2024) who demonstrated that QUE could effectively reduce liver damage by inhibiting the expression of the NLRP3 inflammasome.

NLRP3 inflammasome activation generally includes two steps: priming and activation. The first signal from LPS primes the NLRP3 inflammasome, while another signal from extracellular ROS activates it (Ramachandran et al. 2024). Previous studies have shown that LPS can directly bind to TLR4 on the surface of macrophages, thereby activating the NF‐κB signaling pathway and promoting the synthesis and release of inflammatory mediators (Ciesielska et al. 2021). ROCK belongs to the family of serine/threonine protein kinases that can switch between the active state bound to GTP and the inactive state bound to GDP, acting as molecular switches (Nunes and Webb 2020). p‐MYPT‐1 is a key downstream effector of the ROCK signaling pathway. MYPT1 is the primary substrate of ROCK. Given that ROCK functions as an enzyme, the focus of this research is on its activity rather than its protein expression level. Therefore, this study assessed its downstream substrate rather than ROCK itself. Upon activation, ROCK phosphorylates MYPT1 to form p‐MYPT1, making the expression level of p‐MYPT1 a reliable indicator of ROCK activity (Hartmann et al. 2015). Evidence showed that Rho‐kinase can promote the regulation of NF‐κB (Zhang et al. 2022). Zhao et al. (2023) found that ROCK signaling promoted dysfunction in LPS‐induced sepsis. These suggest that ROCK might be a promising therapeutic target for sepsis caused by LPS. However, whether ROCK mediates the activation of the NLRP3 signaling pathway in SLI has not yet been reported. In this study, we found increased expression of TLR4, p‐MYPT‐1, p‐NF‐κB, and nuclear translocation of p‐NF‐κB in SLI after LPS administration, while these indicators were significantly reduced following QUE pretreatment. Based on these results, we thought that QUE could inhibit the TLR4/ROCK/NLRP3 inflammasome axis to ameliorate the inflammatory response and relieve SLI.

Although our study shares some similarities with previous research on the protective effects of QUE against LPS‐induced organ injury, its novelty lies in the emphasis on the regulatory role of the ROCK/NF‐κB/NLRP3 signaling pathway. We specifically investigated the involvement of ROCK in the progression of SLI and further demonstrated that QUE alleviates SLI potentially by modulating this pathway, offering new insights and theoretical support for its anti‐inflammatory and antioxidant effects. However, this study has limitations. First, the potential crosstalk between ROCK and NLRP3 remains unclear, and whether QUE directly or indirectly inhibits NLRP3 activation requires further cellular investigation. Second, the absence of dose‐ and time‐dependent experimental groups limits understanding of the optimal dosage and timing for QUE's effects. Future studies should explore a broader range of doses and time points. Third, the study primarily used histopathology and biochemical assays, lacking molecular validation techniques such as qPCR. The inclusion of these techniques in future studies would strengthen the reliability of the findings by providing robust molecular evidence.

## 5 Conclusion

In this investigation, our findings demonstrate that QUE has a protective effect against LPS‐induced SLI through a ROCK/NF‐κB/NLRP3‐dependent pathway, at least in part. This pathway involves the interaction between the anti‐inflammatory and antioxidant effects of QUE on SLI rats. These results suggest that QUE could be a therapy for SLI.

## Author Contributions

**Lina Xiao:** writing – original draft (equal). **Lingya Kong:** data curation (equal), software (equal), writing – original draft (equal), writing – review and editing (equal). **Ming Han:** methodology (equal), resources (equal). **Jianing Zhao:** methodology (equal), resources (equal). **Meini Zhang:** data curation (equal), resources (equal). **Yanjin Li:** formal analysis (equal), software (equal). **Mingxuan Wang:** formal analysis (equal), software (equal). **Zirui Wang:** formal analysis (equal), software (equal). **Jiaxuan Li:** formal analysis (equal), software (equal). **Zhihong Ma:** funding acquisition (equal), project administration (equal). **Zhuojun Deng:** conceptualization (equal), funding acquisition (equal), project administration (equal).

## Ethics Statement

This study was approved by the Institutional Animal Care and Use Committee of Hebei University of Chinese Medicine (approval number: DWLL202402041).

## Conflicts of Interest

The authors declare no conflicts of interest.

## Data Availability

The data that support the findings of this study are available on request from the corresponding author.

## References

[fsn370757-bib-0001] Aboyoussef, A. M. , M. K. Mohammad , A. A. Abo‐Saif , and B. A. S. Messiha . 2021. “Granisetron Attenuates Liver Injury and Inflammation in a Rat Model of Cecal Ligation and Puncture‐Induced Sepsis.” Journal of Pharmacological Sciences 147, no. 4: 358–366. 10.1016/j.jphs.2021.08.005. 34663518

[fsn370757-bib-0002] Akbal, A. , A. Dernst , M. Lovotti , M. S. J. Mangan , R. M. McManus , and E. Latz . 2022. “How Location and Cellular Signaling Combine to Activate the NLRP3 Inflammasome.” Cellular & Molecular Immunology 19, no. 11: 1201–1214. 10.1038/s41423-022-00922-w. 36127465 PMC9622870

[fsn370757-bib-0003] Ali, F. E. M. , Z. M. Mohammedsaleh , M. M. Ali , and O. M. Ghogar . 2021. “Impact of Cytokine Storm and Systemic Inflammation on Liver Impairment Patients Infected by SARS‐CoV‐2: Prospective Therapeutic Challenges.” World Journal of Gastroenterology 27, no. 15: 1531–1552. 10.3748/wjg.v27.i15.1531. 33958841 PMC8058655

[fsn370757-bib-0004] Blevins, H. M. , Y. Xu , S. Biby , and S. Zhang . 2022. “The NLRP3 Inflammasome Pathway: A Review of Mechanisms and Inhibitors for the Treatment of Inflammatory Diseases.” Frontiers in Aging Neuroscience 14: 879021. 10.3389/fnagi.2022.879021. 35754962 PMC9226403

[fsn370757-bib-0005] Chen, X. , Y. Liu , Y. Gao , S. Shou , and Y. Chai . 2021. “The Roles of Macrophage Polarization in the Host Immune Response to Sepsis.” International Immunopharmacology 96: 107791. 10.1016/j.intimp.2021.107791. 34162154

[fsn370757-bib-0006] Ciesielska, A. , M. Matyjek , and K. Kwiatkowska . 2021. “TLR4 and CD14 Trafficking and Its Influence on LPS‐Induced Pro‐Inflammatory Signaling.” Cellular and Molecular Life Sciences 78, no. 4: 1233–1261. 10.1007/s00018-020-03656-y. 33057840 PMC7904555

[fsn370757-bib-0007] Dicle, Y. , E. Aydin , and U. Seker . 2024. “Investigation of the Protective Activity of Baicalein on the Lungs via Regulation of Various Cellular Responses in Rats Exposed to Experimental Sepsis.” Toxicological Research (Cambridge) 13, no. 1: tfad112. 10.1093/toxres/tfad112. PMC10762668 38178997

[fsn370757-bib-0008] Ding, R. , J. Han , D. Zhao , Z. Hu , and X. Ma . 2016. “Pretreatment With Rho‐Kinase Inhibitor Ameliorates Lethal Endotoxemia‐Induced Liver Injury by Improving Mitochondrial Function.” International Immunopharmacology 40: 125–130. 10.1016/j.intimp.2016.08.036. 27588912

[fsn370757-bib-0009] Hartmann, S. , A. J. Ridley , and S. Lutz . 2015. “The Function of Rho‐Associated Kinases ROCK1 and ROCK2 in the Pathogenesis of Cardiovascular Disease.” Frontiers in Pharmacology 6: 276. 10.3389/fphar.2015.00276. 26635606 PMC4653301

[fsn370757-bib-0010] Jiang, W.‐L. , X.‐G. Chen , G.‐W. Qu , et al. 2009. “Rosmarinic Acid Protects Against Experimental Sepsis by Inhibiting Proinflammatory Factor Release and Ameliorating Hemodynamics.” Shock 32, no. 6: 608–613. 10.1097/SHK.0b013e3181a48e86. 19295475

[fsn370757-bib-0011] Kannan, S. K. , C. Y. Kim , M. Heidarian , et al. 2024. “Mouse Models of Sepsis.” Current Protocols 4, no. 3: e997. 10.1002/cpz1.997. 38439603 PMC10917121

[fsn370757-bib-0012] Kelley, N. , D. Jeltema , Y. Duan , and Y. He . 2019. “The NLRP3 Inflammasome: An Overview of Mechanisms of Activation and Regulation.” International Journal of Molecular Sciences 20, no. 13: 3328. 10.3390/ijms20133328. 31284572 PMC6651423

[fsn370757-bib-0013] Li, Z. , T. Liu , Y. Feng , et al. 2022. “PPARgamma Alleviates Sepsis‐Induced Liver Injury by Inhibiting Hepatocyte Pyroptosis via Inhibition of the ROS/TXNIP/NLRP3 Signaling Pathway.” Oxidative Medicine and Cellular Longevity 2022: 1269747. 10.1155/2022/1269747. 35136484 PMC8818407

[fsn370757-bib-0014] Liu, H. , Z. Pan , X. Ma , et al. 2022. “ROCK Inhibitor Fasudil Reduces the Expression of Inflammatory Factors in LPS‐Induced Rat Pulmonary Microvascular Endothelial Cells via ROS/NF‐κB Pathway.” BMC Pharmacology and Toxicology 23, no. 1: 24. 10.1186/s40360-022-00565-7. 35428330 PMC9013060

[fsn370757-bib-0015] Liu, Y. , N. Liu , Y. Liu , et al. 2022. “Ginsenoside Rb1 Reduces D‐GalN/LPS‐Induced Acute Liver Injury by Regulating TLR4/NF‐kappaB Signaling and NLRP3 Inflammasome.” Journal of Clinical and Translational Hepatology 10, no. 3: 474–485. 10.14218/JCTH.2021.00072. 35836757 PMC9240244

[fsn370757-bib-0016] Lu, W. , Y. Wang , and J. Wen . 2024. “The Roles of RhoA/ROCK/NF‐kappaB Pathway in Microglia Polarization Following Ischemic Stroke.” Journal of Neuroimmune Pharmacology 19, no. 1: 19. 10.1007/s11481-024-10118-w. 38753217

[fsn370757-bib-0017] Meng, L. , Z. Lv , Z. Z. Yu , D. Xu , and X. Yan . 2016. “Protective Effect of Quercetin on Acute Lung Injury in Rats With Sepsis and Its Influence on ICAM‐1 and MIP‐2 Expression.” Genetics and Molecular Research 15, no. 3: 15037265. 10.4238/gmr.15037265. 27525872

[fsn370757-bib-0018] Miliaraki, M. , P. Briassoulis , S. Ilia , et al. 2022. “Oxidant/Antioxidant Status Is Impaired in Sepsis and Is Related to Anti‐Apoptotic, Inflammatory, and Innate Immunity Alterations.” Antioxidants (Basel) 11, no. 2: 231. 10.3390/antiox11020231. 35204114 PMC8868413

[fsn370757-bib-0019] Nunes, K. P. , and R. C. Webb . 2020. “New Insights Into RhoA/Rho‐Kinase Signaling: A Key Regulator of Vascular Contraction.” Small GTPases 12, no. 5–6: 458–469. 10.1080/21541248.2020.1822721. 32970516 PMC8583239

[fsn370757-bib-0020] Peng, Z. , X. Gong , Y. Yang , et al. 2017. “Hepatoprotective Effect of Quercetin Against LPS/d‐GalN Induced Acute Liver Injury in Mice by Inhibiting the IKK/NF‐kappaB and MAPK Signal Pathways.” International Immunopharmacology 52: 281–289. 10.1016/j.intimp.2017.09.022. 28963941

[fsn370757-bib-0021] Perez‐Hernandez, E. G. , B. Delgado‐Coello , I. Luna‐Reyes , and J. Mas‐Oliva . 2021. “New Insights Into Lipopolysaccharide Inactivation Mechanisms in Sepsis.” Biomedicine & Pharmacotherapy 141: 111890. 10.1016/j.biopha.2021.111890. 34229252

[fsn370757-bib-0022] Puttahanumantharayappa, L. D. , N. G. Sannappagowda , V. D. Shiragannanavar , S. H. Karunakara , and P. K. Santhekadur . 2023. “Ameliorating Effect of Quercetin on Ethanol‐Induced Liver Injury via Targeting RISC Machinery.” International Journal of Health and Allied Sciences 11, no. 3: 1039. 10.55691/2278-344x.1039.

[fsn370757-bib-0023] Ramachandran, R. , A. Manan , J. Kim , and S. Choi . 2024. “NLRP3 Inflammasome: A Key Player in the Pathogenesis of Life‐Style Disorders.” Experimental and Molecular Medicine 56, no. 7: 1488–1500. 10.1038/s12276-024-01261-8. 38945951 PMC11297159

[fsn370757-bib-0042] Sang A. , Y. Wang , S. Wang , Q. Wang , X. Wang , X. Li , X. Song . 2022. “Quercetin attenuates sepsis‐induced acute lung injury via suppressing oxidative stress‐mediated ER stress through activation of SIRT1/AMPK pathways.” Cellular Signalling 96: 110363. 10.1016/j.cellsig.2022.110363. 35644425

[fsn370757-bib-0024] Sannappa Gowda, N. G. , V. D. Shiragannavar , S. H. Karunakara , et al. 2024. “Novel Role of Quercetin in Ameliorating Metabolic Syndrome via VDR Mediated Activation of Adiponectin/AdipoR2 Signaling.” Biochemistry and Biophysics Reports 39: 101754. 10.1016/j.bbrep.2024.101754. 39006943 PMC11246006

[fsn370757-bib-0025] Sannappa Gowda, N. G. , V. D. Shiragannavar , L. D. Puttahanumantharayappa , et al. 2023. “Quercetin Activates Vitamin D Receptor and Ameliorates Breast Cancer Induced Hepatic Inflammation and Fibrosis.” Frontiers in Nutrition 10: 1158633. 10.3389/fnut.2023.1158633. 37153919 PMC10157213

[fsn370757-bib-0026] Seker, U. , D. E. Kavak , F. Z. Dokumaci , S. Kizildag , and S. Irtegun‐Kandemir . 2024. “The Nephroprotective Effect of Quercetin in Cyclophosphamide‐Induced Renal Toxicity Might Be Associated With MAPK/ERK and NF‐κB Signal Modulation Activity.” Drug and Chemical Toxicology 47, no. 6: 1165–1174. 10.1080/01480545.2024.2347541. 38726926

[fsn370757-bib-0027] Seker, U. , E. Uyar , G. S. Gokdemir , D. E. Kavak , and S. Irtegun‐Kandemir . 2025. “The M1/M2 Macrophage Polarization and Hepatoprotective Activity of Quercetin in Cyclophosphamide‐Induced Experimental Liver Toxicity.” Veterinary Medicine and Science 11, no. 1: e70183. 10.1002/vms3.70183. 39792066 PMC11720735

[fsn370757-bib-0028] Singer, M. , C. S. Deutschman , C. W. Seymour , et al. 2016. “The Third International Consensus Definitions for Sepsis and Septic Shock (Sepsis‐3).” Journal of the American Medical Association 315, no. 8: 801–810. 10.1001/jama.2016.0287. 26903338 PMC4968574

[fsn370757-bib-0029] Swanson, K. V. , M. Deng , and J. P. Ting . 2019. “The NLRP3 Inflammasome: Molecular Activation and Regulation to Therapeutics.” Nature Reviews Immunology 19, no. 8: 477–489. 10.1038/s41577-019-0165-0. PMC7807242 31036962

[fsn370757-bib-0030] Tang, X. D. , T. T. Ji , J. R. Dong , et al. 2021. “Pathogenesis and Treatment of Cytokine Storm Induced by Infectious Diseases.” International Journal of Molecular Sciences 22, no. 23: 13009. 10.3390/ijms222313009. 34884813 PMC8658039

[fsn370757-bib-0031] Thomas, R. C. , M. F. Bath , C. M. Stover , D. G. Lambert , and J. P. Thompson . 2014. “Exploring LPS‐Induced Sepsis in Rats and Mice as a Model to Study Potential Protective Effects of the Nociceptin/Orphanin FQ System.” Peptides 61: 56–60. 10.1016/j.peptides.2014.08.009. 25161013

[fsn370757-bib-0032] UludaĞ, Ö. , B. Aydin TÜRk , Z. DoĞAn , et al. 2021. “Sıçanlarda Sepsisin Neden Olduğu Hepatotoksisitede Kuersetin'in Koruyucu ve Terapötik Etkisi.” Kafkas Üniversitesi Veteriner Fakültesi Dergisi 27, no. 6: 699–706. 10.9775/kvfd.2021.26135.

[fsn370757-bib-0033] Vollmannova, A. , T. Bojnanska , J. Musilova , J. Lidikova , and M. Cifrova . 2024. “Quercetin as One of the Most Abundant Represented Biological Valuable Plant Components With Remarkable Chemoprotective Effects—A Review.” Heliyon 10, no. 12: e33342. 10.1016/j.heliyon.2024.e33342. 39021910 PMC11253541

[fsn370757-bib-0034] Wang, W. , L. Ma , B. Liu , and L. Ouyang . 2024. “The Role of Trained Immunity in Sepsis.” Frontiers in Immunology 15: 1449986. 10.3389/fimmu.2024.1449986. 39221248 PMC11363069

[fsn370757-bib-0035] Xu, J. , and G. Nunez . 2023. “The NLRP3 Inflammasome: Activation and Regulation.” Trends in Biochemical Sciences 48, no. 4: 331–344. 10.1016/j.tibs.2022.10.002. 36336552 PMC10023278

[fsn370757-bib-0036] Yuan, R. , H. Xu , M. Wang , et al. 2025. “Promoting the Transition From Pyroptosis to Apoptosis in Endothelial Cells: A Novel Approach to Alleviate Methylglyoxal‐Induced Vascular Damage.” Journal of Translational Medicine 23, no. 1: 170. 10.1186/s12967-025-06195-x. 39930472 PMC11809013

[fsn370757-bib-0037] Zhang, X. , S. Yuan , H. Fan , W. Zhang , and H. Zhang . 2024. “Liensinine Alleviates Sepsis‐Induced Acute Liver Injury by Inhibiting the NF‐kappaB and MAPK Pathways in an Nrf2‐Dependent Manner.” Chemico‐Biological Interactions 396: 111030. 10.1016/j.cbi.2024.111030. 38692452

[fsn370757-bib-0038] Zhang, Y. , X. Qu , H. Gao , et al. 2020. “Quercetin Attenuates NLRP3 Inflammasome Activation and Apoptosis to Protect INH‐Induced Liver Injury via Regulating SIRT1 Pathway.” International Immunopharmacology 85: 106634. 10.1016/j.intimp.2020.106634. 32492628

[fsn370757-bib-0039] Zhang, Y. , X. Wang , C. Jiang , et al. 2022. “Rho Kinase Inhibitor Y27632 Improves Recovery After Spinal Cord Injury by Shifting Astrocyte Phenotype and Morphology via the ROCK/NF‐κB/C3 Pathway.” Neurochemical Research 47, no. 12: 3733–3744. 10.1007/s11064-022-03756-0. 36103106 PMC9718714

[fsn370757-bib-0040] Zhao, L. , J. Hu , P. Zheng , et al. 2023. “PAR1 Regulates Sepsis‐Induced Vascular Endothelial Barrier Dysfunction by Mediating ERM Phosphorylation via the RhoA/ROCK Signaling Pathway.” International Immunopharmacology 124: 110992. 10.1016/j.intimp.2023.110992. 37806106

[fsn370757-bib-0041] Zhao, X. , J. Wang , Y. Deng , et al. 2021. “Quercetin as a Protective Agent for Liver Diseases: A Comprehensive Descriptive Review of the Molecular Mechanism.” Phytotherapy Research 35, no. 9: 4727–4747. 10.1002/ptr.7104. 34159683

